# Adsorption and reversible conformational change of a thiophene based molecule on Au(111)

**DOI:** 10.1038/s41598-023-37661-5

**Published:** 2023-06-30

**Authors:** Suchetana Sarkar, Kwan Ho Au-Yeung, Tim Kühne, Albrecht Waentig, Dmitry A. Ryndyk, Thomas Heine, Gianaurelio Cuniberti, Xinliang Feng, Francesca Moresco

**Affiliations:** 1 Center for Advancing Electronics Dresden, TU Dresden, 01062 Dresden, Germany; 2 Theoretical Chemistry, TU Dresden, 01062 Dresden, Germany; 3 Chair of Molecular Functional Materials and Faculty of Chemistry and Food Chemistry, TU Dresden, 01062 Dresden, Germany; 4 Institute for Materials Science, TU Dresden, 01062 Dresden, Germany

**Keywords:** Nanoscale devices, Nanoscale materials, Organic chemistry, Scanning probe microscopy

## Abstract

We present a low temperature scanning tunneling microscope investigation of a prochiral thiophene-based molecule that self-assembles forming islands with different domains on the Au(111) surface. In the domains, two different conformations of the single molecule are observed, depending on a slight rotation of two adjacent bromothiophene groups. Using voltage pulses from the tip, single molecules can be switched between the two conformations. The electronic states have been measured with scanning tunneling spectroscopy, showing that the electronic resonances are mainly localized at the same positions in both conformations. Density-functional theory calculations support the experimental results. Furthermore, we observe that on Ag(111), only one configuration is present and therefore the switching effect is suppressed.

## Introduction

Advancements in nanoscale scanning probe techniques, such as Scanning Tunneling Microscopy (STM) and Spectroscopy (STS) at low temperatures, allow not only the imaging of molecules on surfaces, but to investigate with a high degree of precision their electronic structure. Moreover, using low temperature STM (LT-STM) we can address individual molecules and study their response to various tip-based manipulations such as inelastic tunneling electrons from the STM tip^1^, electric fields^2,3^ or mechanical manipulation^4^. We are therefore able to experimentally induce and observe conformational changes^5^, rotations^6,7^, translations^8^, and also to trigger chemical reactions on-surface^9–12^, allowing us to gain insights into their underlying mechanisms. In particular, bistable switches on substrates like Si^13,14^, Ge^15^, and metal surfaces^16,17^ show a few examples where conformational changes can be induced by both photo and inelastic excitations towards reversible switching. Furthermore, switching in self-assembled network^2,18^ shows that tip-based manipulation techniques can be used to trigger relatively large scale switching.

**Figure 1:**
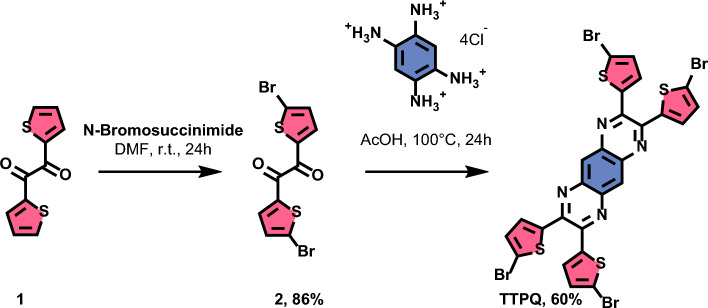
Synthetic route towards TTPQ and chemical structure in gas phase.

Here, we present the study of adsorption geometries and conformational change of a single prochiral^19^ Donor–Acceptor–Donor (D–A–D) type molecule as shown in Fig. 1. The thiophene moieties enhance the donor characteristics of the molecule with a pyrazino[2,3-*g*]quinoxaline backbone as the acceptor core. This design also ensures that rotational flexibility is afforded by the single bonds connecting the four bromothiophene moieties to the rigid backbone.

On Au(111), successful voltage induced flip-flopping of adjacent bromothiophene groups gives rise to switching between two conformational states. Thiophene substituents usually adsorb strongly on metal substrates and do not demonstrate switching behaviour^20^. Density Functional Theory (DFT) calculations and image simulations determining molecular structure and adsorption geometry confirm our results. The role of substrate is found to be important, as on Ag(111), the molecule adsorbs only in one conformation*.* Furthermore, we investigate the electronic resonances and the local densities of states to spatially map charge localization across the two conformers and to clarify the mechanism leading to the switching.

## Molecular structure and synthesis

The molecule, 2,3,7,8-Tetrakis(5-bromo-2-thienyl)pyrazino[2,3-g]quinoxaline (TTPQ) consists of a rigid and planar pyrazinoquinoxaline-core designed as an acceptor and four asymmetric bromothiophene moieties as donor units. TTQP was synthesized in a two-step reaction sequence starting from bromination of **1** with NBS to give dibromothenil **2** in 86% yield. A following imine-condensation of **2** with 1,2,4,5-benzoltetramine-tetrahydrochloride adjusted from a previous report gave the TTPQ as red powder in 60% yield (Fig. 1). Further details and chemical characterization are reported in the Supporting Information file (SI).

## Molecular adsorption on Au(111)

**Figure 2:**
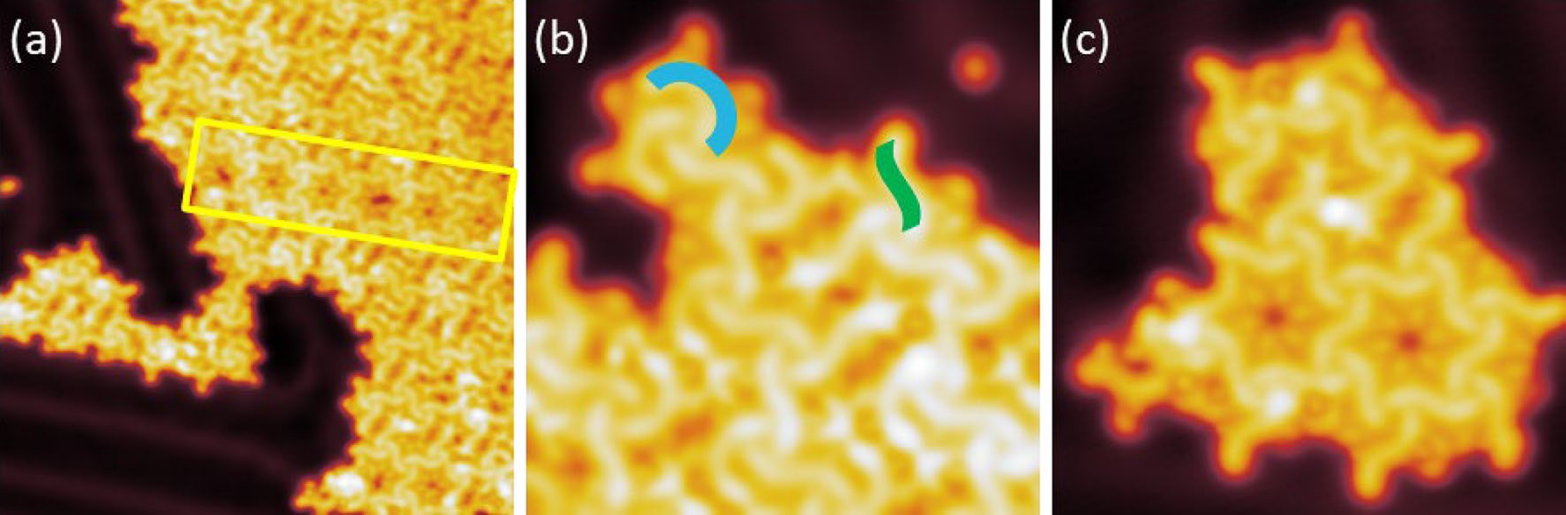
Overview STM images of TTPQ molecules adsorbed on Au(111) after sublimation. (**a**) Self assembled domains with domain wall boundary (yellow rectangle). (**b**) Self-assembled domain formed by a mix of C-Form (in blue) and S-Form (in green) of the molecule, (**c**) Self-assembled domain formed by predominantly S-form of molecule. Image parameters: (**a**) V = 0.5 V and I = 10 pA; 40 nm × 40 nm, (**b**) V = 0.1 V and I = 20 pA; 10 nm × 10 nm, (**c**) V = 0.1 V and I = 20 pA; 12 nm × 12 nm.

TTPQ molecules were sublimated in ultra-high vacuum (UHV) conditions on the Au(111) surface kept at room temperature (RT). After sublimation, the Au(111) sample is transferred to the STM chamber and cooled to 5 K for the STM experiments*. *Figure 2 shows a series of topographic images of the Au(111) surface after the evaporation of molecules. As shown Fig. 2a, the molecules self-assemble in islands, which further enclose different domains as visible by the domain wall boundary (yellow rectangle). In order to investigate the arrangement of the molecules in these domains, smaller area STM images were recorded, which are shown in Fig. 2b,c. Figure 2b shows that the molecule adsorbs on the surface in two distinct conformations, one is dubbed the C-form (Fig. 2b in blue), and the other is called the S-Form (Fig. 2b in green). Therefore, this particular domain is formed by the close packed arrangement of both conformations. Figure 2c, on the other hand, shows a different domain structure as it is formed predominately by the S-form of the molecule. Figure 2b,c have features which appear as dots that we assign to bromine atoms*.* Larger islands exhibit better long-range order and are formed by both forms of the TTPQ molecules. In the SI we have considered one of these islands and determined the rate of C and S-Forms as well as the periodic parameters (Fig. S8). Notably, in all observed islands, the molecules are homochiral. We also note that isolated molecules are vanishingly rare.

## Lateral manipulation via STM tip

**Figure 3:**
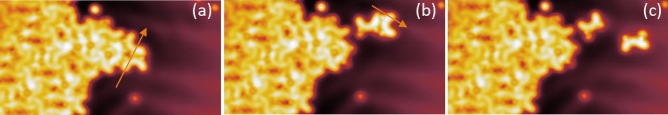
Lateral manipulations of a single molecule from an island. (**a**) STM image of an ordered molecular assembly. Lateral manipulation is applied (V = 10 mV, I = 2 nA), where the arrow indicates the trajectory of the STM tip. (**b**) Separation of two molecules from the island and further lateral manipulation using the same parameters is applied along the path shown by the arrow. (**c**) Completely isolated molecules, both of the C-form. Image parameters: V = 0.5 V and I = 10 pA; 20 nm × 8 nm.

In order to investigate single molecules, lateral manipulation was performed on the island edges. This is a well-documented technique wherein the STM tip is positioned over the user-determined starting point and then moved along a specific trajectory under constant current mode^4,21,22^. The molecules follow the manipulation path and the typically low voltage setting ensures that this technique is non-destructive and reproducible. In the sequence shown in Fig. 3, the edge terminus is formed by two C-form molecules that were successfully separated first from the island (Fig. 3a) and then from each other (Fig. 3b), giving two isolated molecules (Fig. 3c). In some cases, like the one shown above in Fig. 3c, the single molecules have undergone partial debromination while in other cases shown in the upcoming sections, all bromines are found to be intact. Characteristic lateral manipulation curves along the arrow can be found in the SI (Fig. S9).

## Single molecule investigation

**Figure 4:**
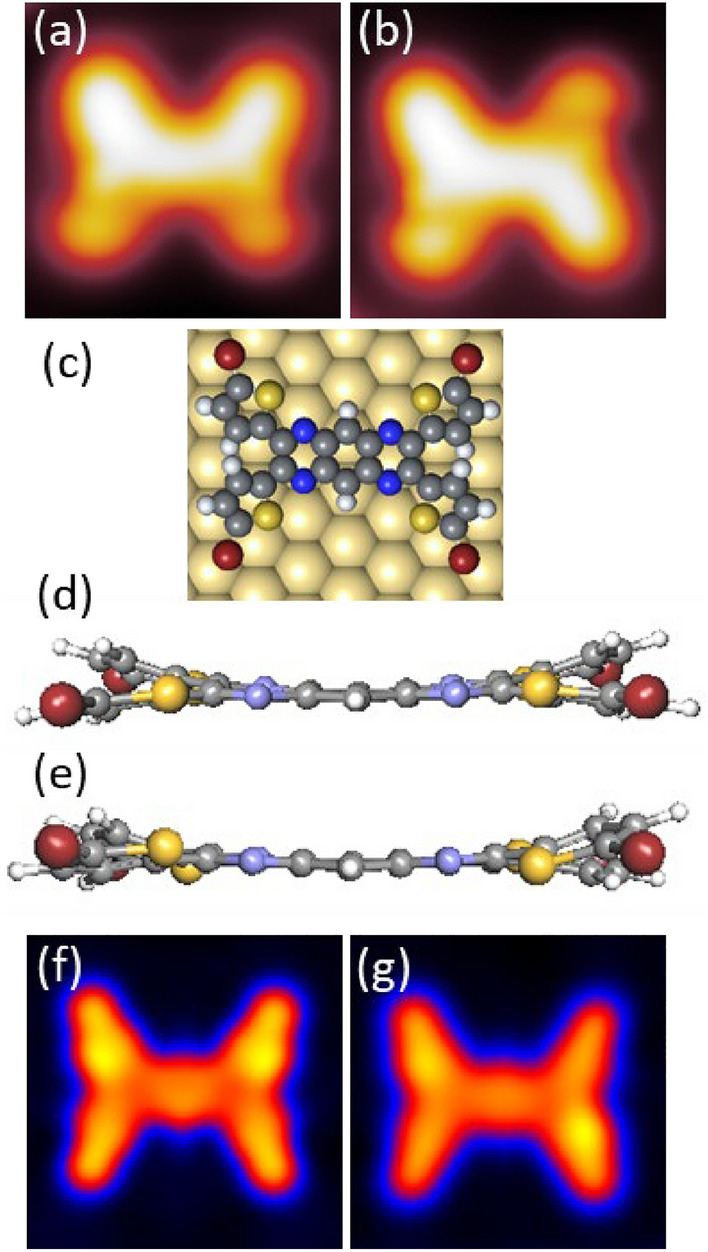
(**a**, **b**) Experimental STM images of C-Form and S-Form, respectively. Image Parameters: V = 0.1 V and I = 10 pA; 3.0 nm × 3.0 nm. DFT calculations of adsorption geometry of TTPQ on Au(111): (**c**) Top view of adsorption geometry, (**d**) Molecular structure showing cis symmetry (C-Form), (**e**) Molecular structure showing the trans symmetry (S-Form). (**f**,**g**) Simulated STM images of C-Form and S-Form, respectively.

Figure 4a,b show the STM images of isolated and fully brominated C-Form and S-Form (respectively) after separation from an island. The adsorption geometry of the single molecule on the Au(111) surface was calculated by DFT. The energetically most favourable form was found to be the one with all sulphur atoms of the four bromothiophene groups pointing outwards as shown in Fig. 4c. This is in contrast with the gas phase structure shown in Fig. 1*,* since the surface usually acts as a constraint and limits the degrees of freedom that are present in gas phase. To understand the origin of the two molecular forms observed on the surface, one must look in detail at the adsorption geometry of the bromothiophene side groups. Figure 4d shows the simulated C-form (or the cis conformer), which is obtained when the top two side groups in Fig. 4c point towards the surface and, due to steric hindrance of the adjacent hydrogen atoms, the bottom two bromothiophenes are inevitably closer to the surface. Figure 4e shows the simulated S-form (or trans conformer), where one of the top bromothiophenes is adsorbed closer to the surface, while the other points away, and the adjacent bottom bromothiophene adsorption is dictated by steric hindrance. Figure 4f,g is the corresponding calculated STM images of the C and S-form respectively, based on the aforementioned geometries. Further adsorption geometries and calculated images can be found in the SI (Fig. S5). The calculated and experimental images are in good agreement, which helps in concluding that the two observed conformations of TTPQ on the Au(111) surface can be attributed to a slight rotation of two adjacent bromothiophene groups.

## Electronic properties

**Figure 5:**
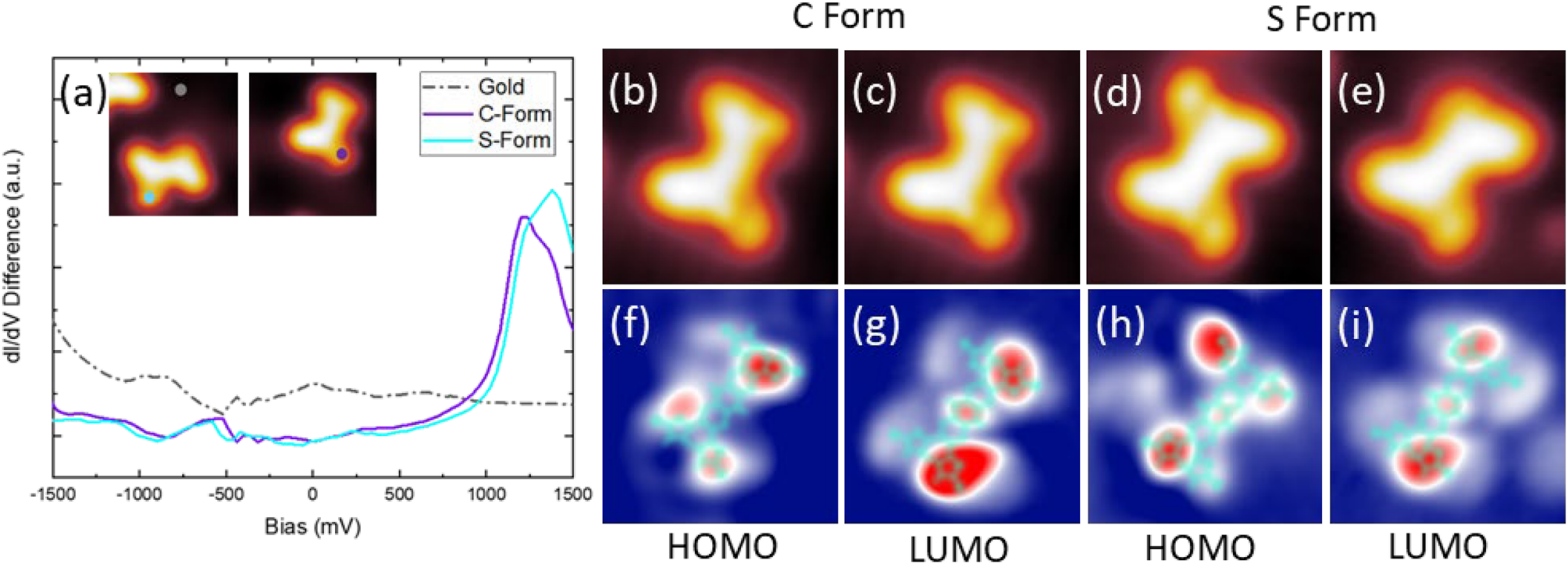
(**a**) STS spectra of both forms of the molecule (Inset). (**b**–**e**) Topographical STM images (V = 0.5 V and I = 20 pA), with (**d**) having all Br intact. (**f**–**i**) Corresponding dI/dV maps taken in constant current mode at (**f**) − 0.6 V, (**g**) 1.2 V, (**h**) − 0.6 V, (**i**) 1.2 V All images 3.5 nm × 3.5 nm.

To determine the electronic resonances of the molecule and characterize its electronic structure, STS was performed. Figure 5a shows the dI/dV spectra of both forms of the molecule and (in the inset) the position at which the spectra were taken. Below the Fermi energy, both conformers show slight offset with respect to each other, the first peak around − 0.6 V, which we ascribe to the last occupied molecular resonance (for simplicity called Highest Occupied Molecular Orbital (HOMO)). Above the Fermi energy, two resonances are visible at 1.2 V for the C-form and due to a shift of 0.2 V, at 1.4 V for the S-Form. This shift seen at both HOMO and LUMO resonances can be attributed to the difference in conformations^[2]^. However, the peaks are relatively broad, probably indicating that the tunneling electron resonance is formed by the superposition of several peaks. We denote the resonance peak at 1.2 V as Lowest Unoccupied Molecular Orbital (LUMO). With this ascription of the frontier molecular orbitals, we arrive at a bandgap of 1.8 V, which falls within the range of typical D-A-D molecules^23,24^.

Figure 5f–i shows differential conductance maps recorded at the resonances discussed previously, i.e., at − 0.6 V and 1.2 V. Superimposing the chemical structure on the conductance maps (Fig. 5f–i in neon blue) and following the corresponding STM topographies shown in Fig. 5b–e allows us to discern where the electronic densities are localized. As shown in Fig. 5g,i, there is a localization of LUMO resonances on the bromothiophenes that are in plane with the pyrazinoquinoxaline-core. In other words, around the sulphur moieties that are shifted away from the Au(111) surface (differentiated lobes in the topography shown in Fig. 5c) as compared to the sulphur moieties that are closer to the surface (maxima in the topography shown in Fig. 5e). The spatial localization of the HOMO resonance also appears to be restricted to the bromothiophene groups but is less affected by the exact orientation of the thiophene-moiety (Fig. 5f,h). We also observe a contribution of the central quinoxaline ring to the contrast in all maps. The slight change in STM topography between the images is due to the fact that in Fig. 5d all bromines are intact, while in the other cases a bromine is dissociated (see SI Fig. S11 for an example of tip-induced Br dissociation). The Br dissociation does not affect energy or spatial localization of the electronic resonances.

## Voltage pulse induced switching

**Figure 6:**
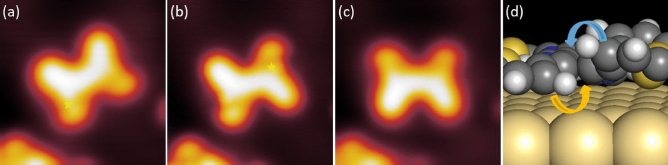
On-surface reversible switching event of the C-form of molecule. (**a**) A voltage pulse (V = 2.0 V, 5 s; marked position) is applied above the molecule that was isolated. (**b**) A slight lateral displacement was induced, and the appearance of the molecule has changed to the S-form. (**c**) Upon a further pulse in the marked position, the molecule switches back to C-form. Image parameters: V = 0.1 V and I = 20 pA; 6 nm × 6 nm. (**d**) DFT calculation of the bromothiophene groups. The arrows indicate the rotational direction.

The two distinct conformations of the molecule on the Au(111) surface led us to explore the possibility of switching the molecule. In order to do so, we positioned the tip above the molecule and applied a voltage pulse of 2 V for 5 s. This technique induces tunneling electrons to travel from the tip to the sample, and a small percentage of these can inelastically excite the molecule to induce conformational changes^7^. Figure 6 shows a sequence where we successfully changed the molecule from C-form to S-form and then back to C-form again. As shown in Fig. 6a*,* the tip is first placed above the C-form of the molecule, specifically, over one of the bromothiophene groups (in the spot designated with the star) and then pulsed. Upon applying the voltage pulse in constant height mode, a sudden current drop is recorded, indicating a switching event (see SI Fig. S10). We find this pulse to have triggered the rotation of the opposite bromothiophene group, as shown in Fig. 6b. The next pulse is applied to the switched bromothiophene group shown in Fig. 6b, and we record a similar current drop indicating that another switching event has occurred. The next STM topography shown in Fig. 6c confirms this, where we find that the opposite leg has switched, producing the C-form again.

Nudged Elastic Band (NEB) transition state searches show that the barrier for going from S-form to C-form is around 0.08 eV while from C-form to S-form is around 0.04 eV (SI Fig. S6), confirming that the two conformations are very close in energy and that a switch is possible.

The voltage required to trigger the switching lies beyond the LUMO resonance of 1.2 V, indicating that the observed switching is driven by inelastic electron tunneling through electronic excited states. In inelastic electron tunneling manipulation, part of the tunneling electron energy is transferred to the molecule through excited states, leading to rotational, vibrational or electronic excitations^25^. The molecule then relaxes transforming this excitation energy in movement on the surface or, in the present case, in an intramolecular conformational change. The position on the molecule where the voltage pulse is applied does not necessarily correspond to the part of the molecule which is going to move or to change configuration after relaxation, but more to the position where the electronic or vibrational mode can be excited^8^. In this specific case, the LUMO localization around the raised sulphur moieties coincides with the pulse location for successful events, further confirming the excitation of electronic resonances. This is also in agreement with the LUMO calculations in Gas phase (see SI Fig. S7), which show two symmetric charge localization lobes around the bromothiophenes.

We conclude that by pulsing on the raised sulphur moieties, we induce its tilting towards the surface, with the rotation of the adjacent bromothiophene group due to steric hindrance. This mechanism can be better understood graphically by following Fig. 6d. This is a close-up of the calculated adsorption geometry of the TTPQ molecule, focusing on the sideview of the bromothiophene groups. The yellow and blue arrows indicate the flip-flopping of the adjacent hydrogen atoms. This explains the lack of intermediate switched structures, since it is always the reciprocal change in the adsorption site of the bromothiophene groups that causes the switching.

## Role of substrate: Molecular adsorption on Ag (111)

**Figure 7:**
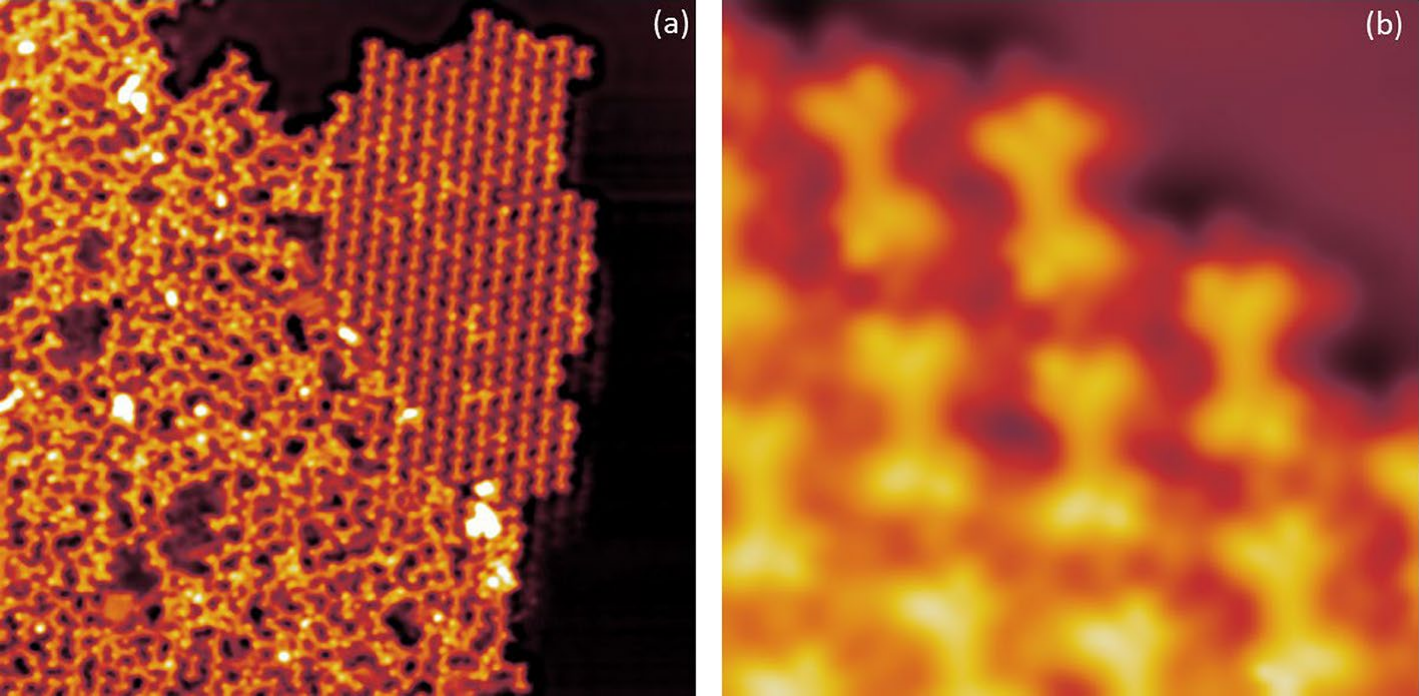
(**a**) Self-assembly upon evaporation onto RT Ag(111) showing linkages and single molecules. (**b**) Single molecules surrounded by dissociated bromines. Image parameters: V = 0.5 V and I = 10 pA; (**a**) 40 nm × 40 nm, (**b**) 5 nm × 5 nm.

Figure 7 presents the adsorption of TTPQ molecules on the Ag(111) surface. Figure 7a shows a large area STM image where disordered structures of linked TTPQ molecules can be observed in close proximity to an island of separated molecules. Figure 7b shows a closer look at the island. As one can see, the molecules are surrounded by dissociated bromines. It is well-reported in literature that halogenated molecules can be dehalogenated upon RT adsorption on silver surfaces due to its enhanced catalytic nature^26^. Indeed, we observe the same upon evaporating the TTPQ molecules onto Ag(111) kept at RT. Furthermore, differently from the Au(111) case, all the molecules appear in the same conformation and therefore cannot be switched.

## Conclusions

We have presented the LT-STM studies of TTPQ molecules adsorbed on two metals substrates, viz., Au(111) and Ag(111). On Au(111), we studied the adsorption, reversible conformer switching, and the electronic properties of the molecule. By applying STM voltage pulses, we have shown that one conformer can be changed to the other. The mechanism responsible for this switching is probably related to the electronic excited states addressed by the tunneling electrons. The calculated adsorption geometries demonstrate the pathways in which the bromothiophene groups can alternate between two stable states. Differently from the case of the Au(111) surface, the adsorption geometry of the molecules on Ag(111) is restricted to only one form. As an outlook, we notice that different possibilities of donor acceptor atoms can further dope the molecule, leading to a narrower bandgap, thereby tuning its optoelectronic properties.

## Methods

The TTPQ molecules were evaporated at 280 °C for 30 s on an Au(111) surface kept at room temperature (25 °C) and deposited similarly on Ag(111). Before evaporation, the samples were cleaned by subsequent cycles of Ar^+^ sputtering and annealing to 450 °C. STM experiments were performed using a custom-built instrument operating at a low temperature of *T* = 5 K under ultrahigh vacuum (*p *≈ 1 × 10^−10^ mbar). All STM images were recorded in constant-current mode with the bias voltage applied to the sample.

In order to probe the voltage pulse induced switching of the molecule, the STM tip was positioned at a fixed height above a molecule (non-contact) with the feedback loop switched off and a voltage *V* was applied within a fixed time window. STM images were recorded both before and after each pulsing event.

All lateral manipulations were performed in constant-current mode. The lateral manipulation procedure involves three steps: (1) allowing the tip to vertically approach the molecules under a small bias and current to increase the tip–molecule interaction, (2) laterally driving the tip parallel to the surface in a precisely controlled trajectory, and (3) retracting the tip to normal scanning position. The STM captures images before and after each manipulation.

For geometry optimization, reaction path calculations, and STM images we used the DFT as implemented in the CP2K software package^27^ (cp2k.org) with the Quickstep module^28^. We applied the Perdew-Burke-Ernzerhof exchange–correlation functional^29^, the Goedecker-Teter-Hutter pseudo-potentials^30^ and the valence double-ζ basis sets, in combination with the DFT-D2 method of Grimme^31^ for van der Waals correction. We used 6 layers of gold, 3 upper layers allowed to be relaxed, planar supercell 29.8 × 19.9 Angstrom, vacuum size 40 Angstrom, maximum force 4.5 × 10^–5^ a.u. The data is analysed, and the images are made by the PyMOL Molecular Graphics System, Version 2.4 open-source build, Schrödinger, LLC.

## Supplementary Information

Supplementary Information.

## Data Availability

The datasets used and/or analysed during the current study available from the corresponding author on reasonable request.
