# Supplementary material for: Adsorption and reversible conformational change of a thiophene based molecule on Au(111)
Source: Sci Rep. 2023 Jun 30;13:10627. doi: 10.1038/s41598-023-37661-5 (PMC10313653; doi:10.1038/s41598-023-37661-5)
Supplement: Supplementary file 1 — Supplementary Information. [file 41598_2023_37661_MOESM1_ESM.pdf]

Adsorption and reversible conformational change of a thiophene based molecule on Au (111)

Suchetana Sarkar[1], Kwan Ho Au-Yeung[1], Tim Kühne[1], Albrecht Waentig[1,3], Dmitry A. Ryndyk[2], Xinliang Feng[1,3], Francesca Moresco[1]*

[1] Center for Advancing Electronics Dresden, TU Dresden, 01062 Dresden, Germany

[2] Theoretical Chemistry, TU Dresden, 01062 Dresden, Germany

[3] Chair of Molecular Functional Materials and Faculty of Chemistry & Food Chemistry, TU Dresden, 01062 Dresden, Germany

* Email: francesca.moresco@tu-dresden.de;

Table of Contents

General information 3

Synthesis of 1,2-Bis(5-bromo-2-thienyl)-1,2-ethanedione (2) 3

Synthesis of 2,3,7,8-Tetrakis(5-bromo-2-thienyl)pyrazino[2,3-g]quinoxaline (TTPQ) 3

Characterization of TTQP 4

Mass spectroscopy 4

NMR spectroscopy 5

TGA-FTIR Data 5

DFT Calculations 6

NEB Calculations 7

Gasphase Calculation of LUMO 7

Additional overview STM image 8

Isolation of single molecule 9

Additional data of switching molecule C-Form to S-Form 10

Pulse-induced Debromination 11

Post-Annealing Results 12

General information

Commercially available reagents and solvents were used without further purification unless otherwise stated. The reactions were performed using standard Schlenk techniques, purification and workup of all compounds was performed under ambient conditions with reagent-grade solvents. Silica coated aluminum sheets with fluorescence indicator from Merck were used for the thin-layer chromatography.

Nuclear magnetic resonance (NMR) spectroscopy: 1^{H}, 13^{C}- NMR spectra were recorded on a BRUKER AVANCE III 300 at 25°C using standard pulse programs. Chemical shifts are reported as δ-values in ppm. The coupling constants (J) are given in Hertz (Hz). The chemical shift was referenced in regards to δ chloroform-d1 (δ (1H) = 7.26 ppm, δ (13C) = 77.16 ppm). To describe peak patterns, following abbreviations were used: s = singlet, d = doublet, m = multiplet.

Mass spectrometry (MS): For mass spectra, a Bruker Autoflex Speed MALDI-TOF using dithranol as a matrix in positive mode was employed. Atmospheric Pressure Chemical Ionization (APCI) mass spectra was recorded with Agilent 6538 Ultra High Definition (UHD) Accurate-Mass QTOF LC/MC system, using the positive mode.

Synthesis of 1,2-Bis(5-bromo-2-thienyl)-1,2-ethanedione (2)

1,2-Di-2-thienyl-1,2-ethanedione (**1**) (2g, 9 mmol, 1 eq.) were dissolved in 50 mL degassed dimethylformamide under nitrogen in a 250 mL Schlenk flask. 5.61 g N-Bromosuccinimide (31.5 mmol, 3.5 eq.) was added and the mixture was stirred in the dark for 24 h at 25°C under nitrogen atmosphere. After full conversion (progress studied by thin layer chromatography (isohexane+ DCM)), the reaction mixture was poured in 100 ml of cold water. The precipitate was collected and thoroughly washed with water and cold methanol. After repeated recrystallization from hexanes, compound **2** was obtained as a yellow (2.91 g, 86% yield).

NMR spectroscopy and MS of **2** were in good accordance with previous reports **[1,2]**.

Synthesis of 2,3,7,8-Tetrakis(5-bromo-2-thienyl)pyrazino[2,3-g]quinoxaline (TTPQ)

Adapted from[1], 1,2-Bis(5-bromo-2-thienyl)-1,2-ethanedione (**2**) (0.2 g, 0.53 mmol, 1.5 eq.) and 1,2,4,5-Tetraaminobenzene tetrahydrochloride (100 mg, 0.35mmol, 1 eq.) were dissolved in 3.5 mL concentrated acetic acid. The mixture was degassed by nitrogen flushing and heated for 24 h at 100 °C. The red precipitate was washed with water, acetone and methanol. 170 mg

(yield: 60%) **TTQP** were obtained as a red powder. Solubility in different solvents was not sufficient for ^{13}C-NMR spectroscopy.

1H NMR (300 MHz, $C_2D_2Cl_4$) δ 8.65 (1H) 7.24 (d, J = 4.0 Hz, 2H), 7.04 (d, J = 4.0 Hz, 2H)

APCI-MS: m/z (M+H) = 826.66, calc. For $C_{26}H_{11}Br_4N_4S_4$: m/z = 826,66

MALDI-TOF-MS: m/z (M+H) = 826.7, calc. For $C_{26}H_{11}Br_4N_4S_4$: m/z = 826,66

Characterization of TTQP

Mass spectroscopy

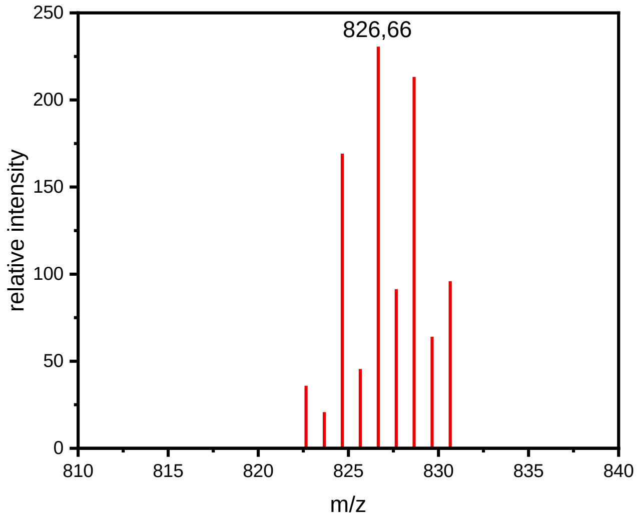

Figure S1: APCI-MS spectrum of **TTQP**

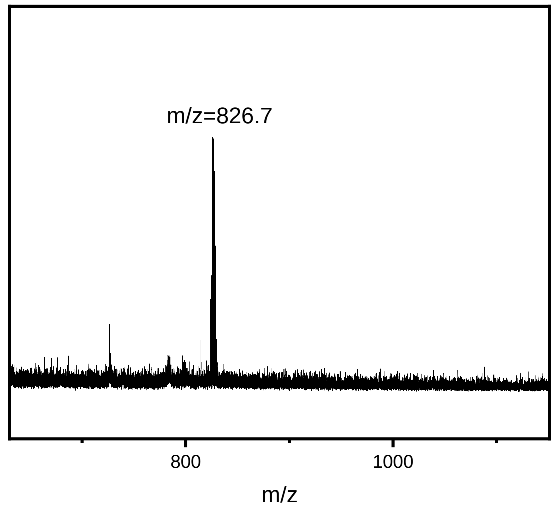

Figure S2: Full Maldi-TOF spectrum of **TTQP**

NMR spectroscopy

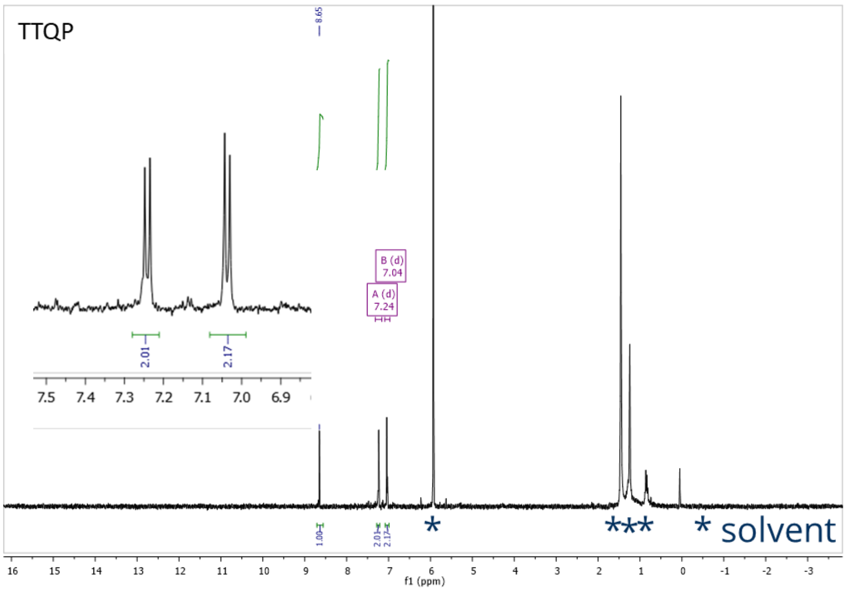

Figure S3: 1H-NMR spectrum of **TTQP**

TGA-FTIR Data

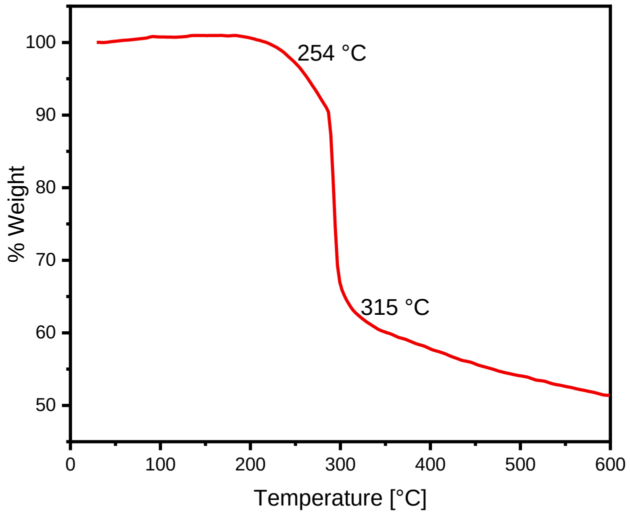

Figure S4. Weight loss of Molecule by increasing the temperature.

DFT Calculations

Figure S5a-c shows different adsorption geometries calculated by DFT. The calculated STM images are shown in Figure S5d-f. As discussed in the main text, experimental results in agreement with calculated images confirm the correct adsorption geometry.

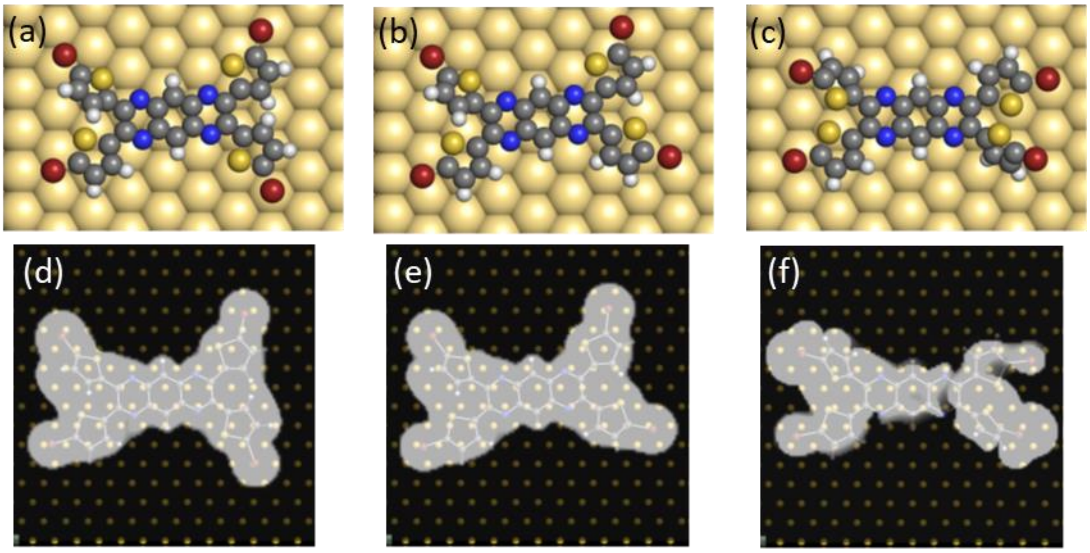

Figure S5. (a)-(c) DFT calculated adsorption geometries showing different conformations of the bromothiophene groups. (d)- (f) Calculated STM images

NEB Calculations

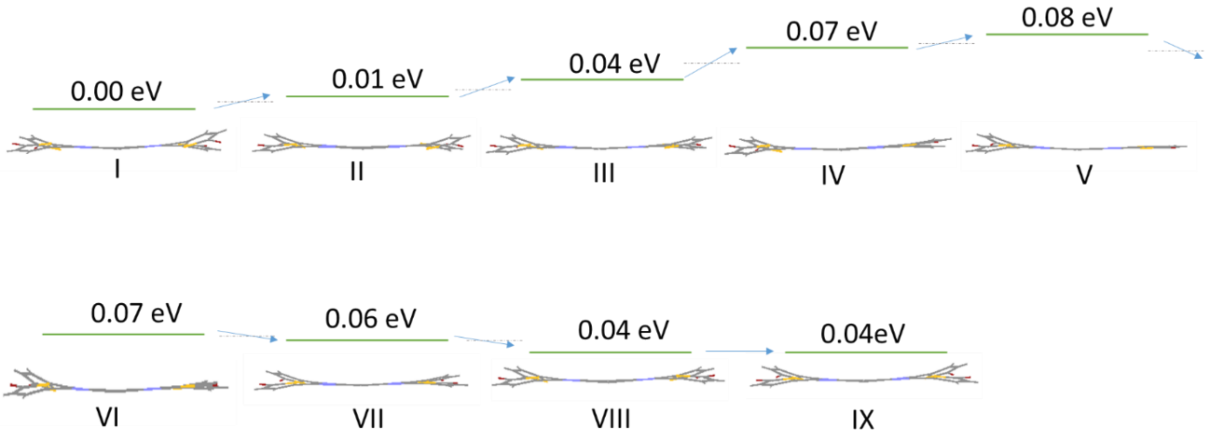

Figure S6. Energy profiles at different states and the corresponding adsorption geometries. Side view of the simulated adsorption geometries, calculated by DFT, starting from the S-Form (state I), through intermediate states (II–VIII) to the C-Form (state IX). The corresponding reaction energy profiles (in eV) are calculated by the nudged elastic band (NEB) method.

Gasphase Calculation of LUMO

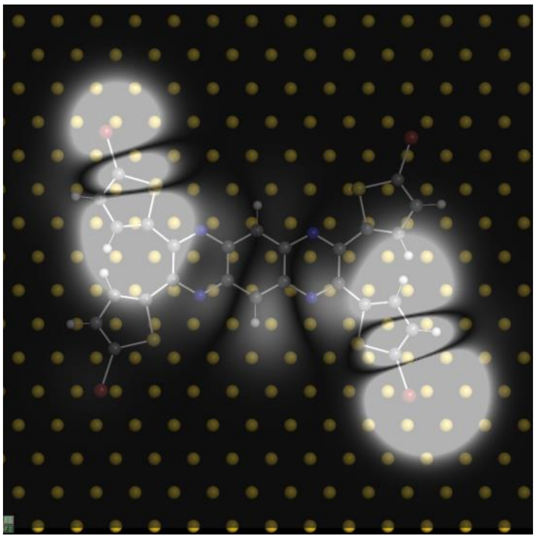

Figure S7: Calculation of the LUMO orbitals of the S-Form of TTPQ molecule in gasphase

Additional overview STM image

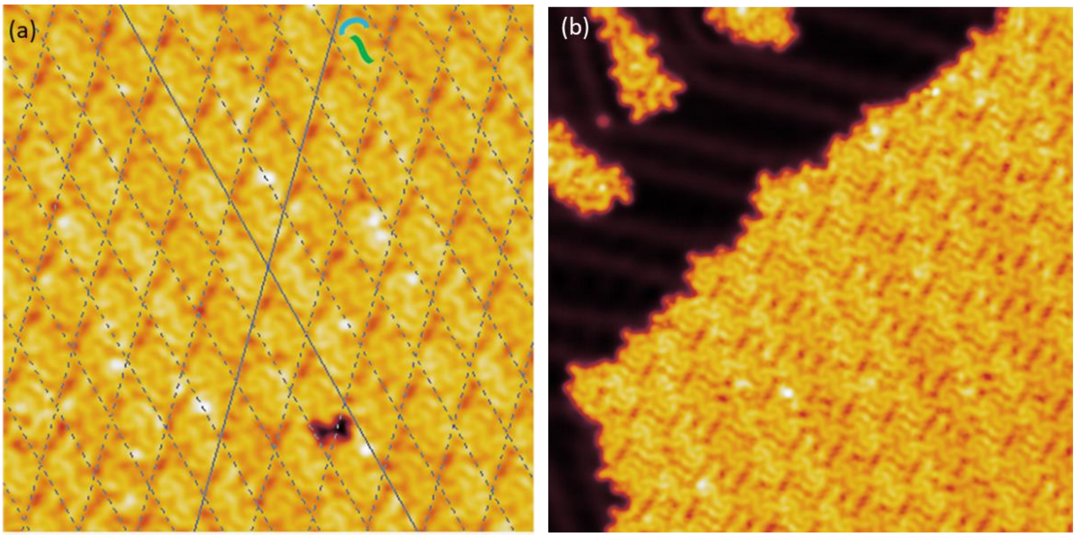

	x[nm]	y[nm]	Length[nm]	Angle[deg]
a_1	0.9	3.3	3.4	74.5
a_2	-2.2	3.9	4.4	119.4

ϕ: 44.9

Figure S8. Additional overview STM topography of self-assembly of TTPQ with lattice parameters. (a) Mixed TTPQ molecule island with S(shown in green) and C(shown in blue) forms. The overlay shows the unit cell used calculate the values shown in the table. (b) Larger island with the same periodic parameters. STM images were obtained under the conditions of $V = 0.2$ V and $I = 20$ pA. Image size: (a) 30nm x30nm (b) 80nm x80nm

In order to calculate the periodic parameters of the self-assembled islands, an exemplary island formed by both S and C form was chosen, as shown in Figure S8. Smaller islands do not exhibit discernible long-range order. As shown in Figure S8 (a), we determined the unit cell of an island with long-range order, thus obtaining the lattice constants that are listed in the table. Each unit cell is formed by two C-form molecules and at least three S-form molecules. The inset of green and blue in the same figure is a guide to the eye for convenience. Figure S8 (b) shows a larger island with the same configuration. Assuming the unit cell to be accurate for all observed large islands, we arrive at the S-form being slightly more populous at 57% and the C-form at 43%.

Isolation of single molecule

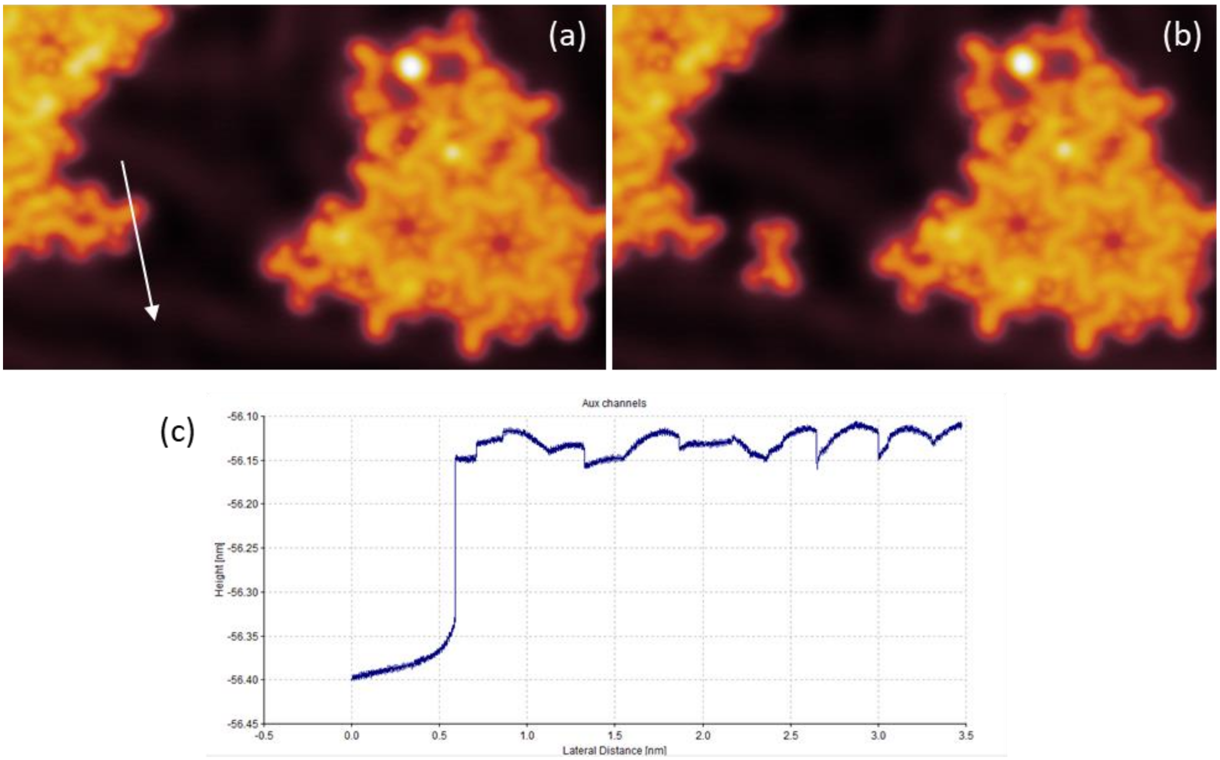

Figure S9. Isolation of single molecule **(C-Form)** from the edge of the molecular island. (a) White arrow in the manipulation sequence indicate trajectories of lateral manipulations in constant current mode (V = 10 mV; I = 2 nA). (b) The subsequent image shows that the molecule has been successfully isolated from the island, following the trajectory of the tip. (c) Tip behavior during manipulation showing tip height vs lateral distance curve. STM images (20 nm x 12 nm) were obtained under the conditions of V = 0.1 V and I = 50 pA.

Additional data of switching molecule C-Form to S-Form

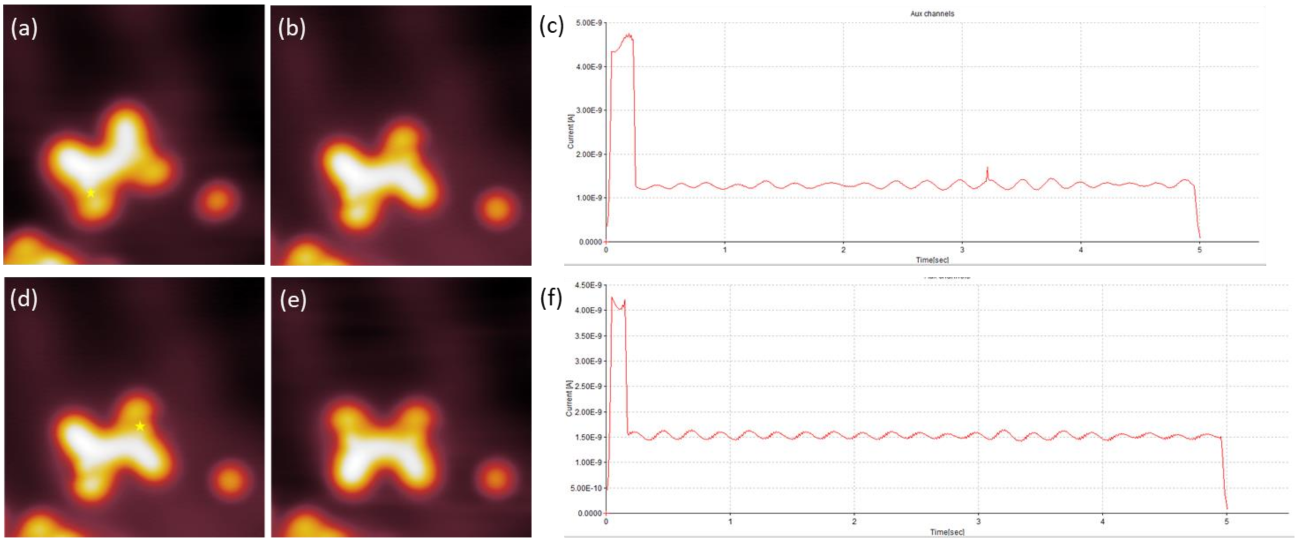

Figure S10. STM voltage pulse of 2V induced switching of a single molecule. (a)- (b) Switching from C-Form to S-Form. (c) Current vs. time curve showing tip behavior during switching. (d)-(e) Switching from S-Form to C-Form and (e) Tip behavior during switching even shown in All STM images (6 nm × 6 nm) were obtained under the conditions of V = 0.1 V and I = 10 pA.

Pulse-induced Debromination

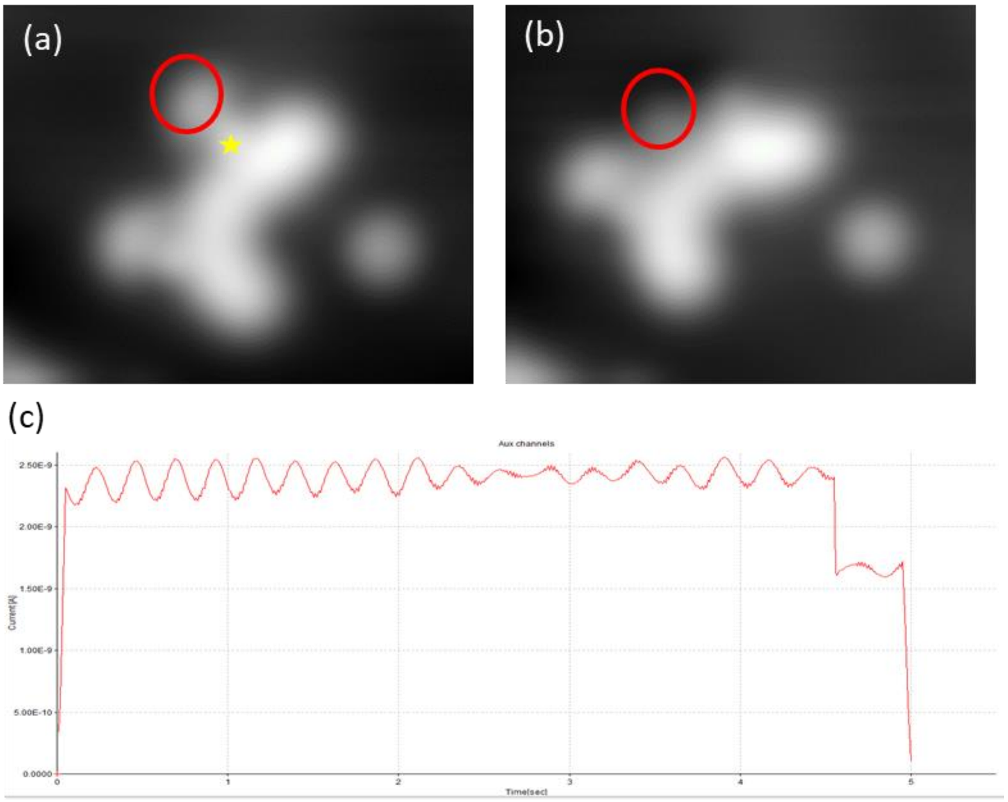

Figure S11. STM voltage pulse induced debromination of single molecule. Pulse Parameters: 2.2V/5seconds. All STM images (5nm × 4 nm) were obtained under the conditions of V = 0.1 V and I = 10 pA

We occasionally observe debromination events, an example of which is shown above. Bromines are found to cleave during pulses of 2.2V and higher and also during high bias measurements.

Post-Annealing Results

Post-annealing to the temperatures shown in the figure for 15 minutes leads to debromination and linkages without long-range order.

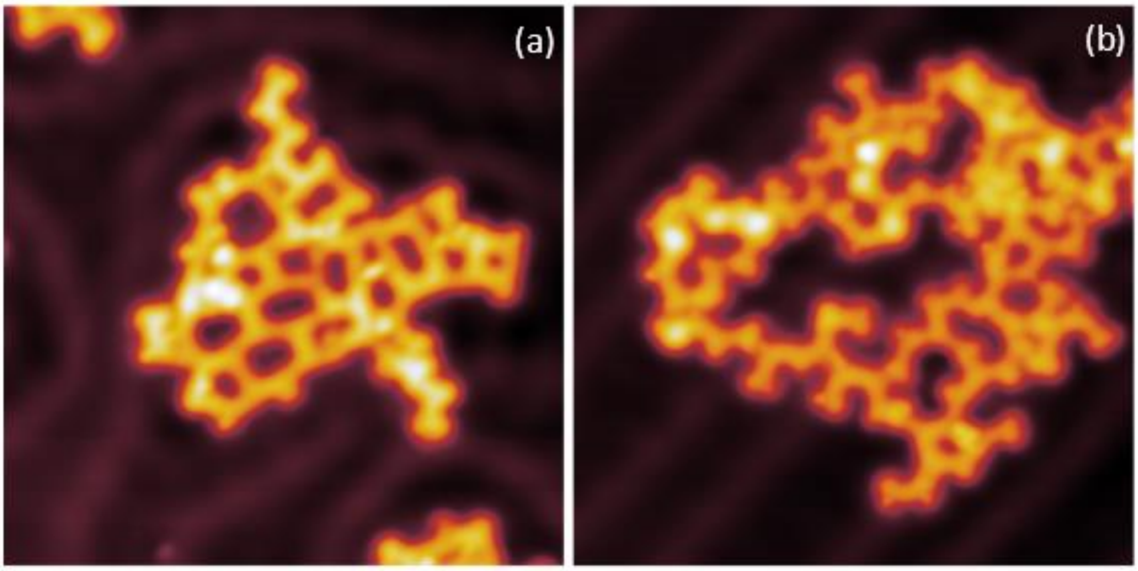

Figure S12. Post-annealing TTPQ molecules (a) 200C (b) 250C. All STM images (6 nm × 6 nm) were obtained under the conditions of V = 0.1 V and I = 10 pA.

References

1 Baeuerle, P *et. al.*, Conjugated Oligothienyl Dendrimers Based on a Pyrazino[2,3-*g*]quinoxaline Core, Org. Lett., **11**, 20, 4500–4503(2009)

2 Journal of Polymer Science, Part A: Polymer Chemistry, **51**, 1565–1572, (2013)
